# Supplementary figures and images for: Psychometric Properties of the 11-Item De Jong Gierveld Loneliness Scale in a Representative Sample of Mexican Older Adults
Source: Healthcare (Basel). 2023 Feb 8;11(4):489. doi: 10.3390/healthcare11040489 (PMC9957099; doi:10.3390/healthcare11040489)

Supplementary Figure S1: Scree Plot

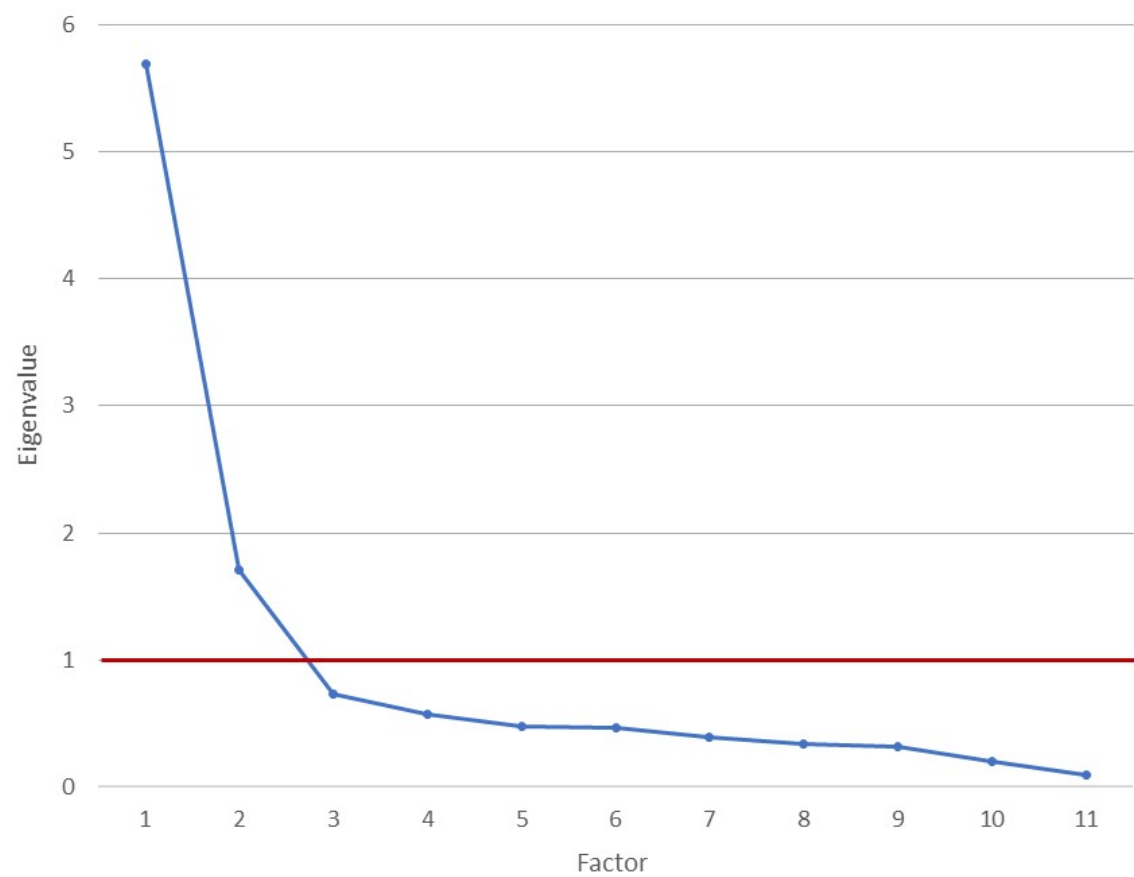

Supplement: Supplementary file 1 [file healthcare-11-00489-s001.zip › healthcare-2006460-supplementary.pdf]
